# “I am because we are”: Ubuntu as a framework for social capital building among Black Women in the academy

**DOI:** 10.3389/fsoc.2025.1555236

**Published:** 2025-07-02

**Authors:** Chiedza Ikpeh, Iman Federico Awi

**Affiliations:** ^1^School of Education, University of Chester, Chester, United Kingdom; ^2^School of Sociology and Social Policy, University of Leeds, Leeds, United Kingdom

**Keywords:** Ubuntu, social capital, Black Women, higher education, equity, mentorship, informal networks, epistemic justice

## 1 Introduction

Ubuntu, an African philosophy centered on interconnectedness and mutual care, offers a transformative framework for addressing systemic inequities in academia (Dillard and Neal, 2020; Letseka, 2012). Its ethos is the interdependence of individuals and the importance of fostering shared humanity to uplift all members of a community (Mbigi and Maree, 1995; Letseka, 2012). This philosophy challenges the individualistic paradigms that dominate many academic environments, particularly in the West (Metz, 2017).

Research continues to evidence that Black Women face significant systemic barriers in the academy, including underrepresentation in faculty and leadership roles, exclusion from traditional mentorship networks and limited access to professional opportunities (Patton and Catching, 2009; Collins, 2000). In 2022–2023, only 0.2% of professors in the UK were Black Women, with just 60 out of 24,405 professor positions held by them (HESA, 2024). These stark disparities underscore the urgent need for Ubuntu-inspired approaches to academic equity.

This paper proposes that embedding Ubuntu's principles into higher education policies and structures could foster mentorship, collaboration, and social capital, creating equitable networks that empower marginalized voices. Ubuntu's holistic approach addresses inequities while promoting inclusivity and collective progress, paving the way for systemic transformation in higher education.

### 1.1 The philosophical foundations of Ubuntu

Ubuntu, as a philosophy, is inherently counter-hegemonic, resisting Western paradigms that prioritize individual agency over collective responsibility. Unlike Western frameworks that emphasize binary oppositions—self vs. other, rational vs. emotional—Ubuntu embraces relationality as the foundation of existence (Robinson-Morris, 2018). While equity remains a structural principle, Ubuntu extends beyond institutional frameworks to reshape human interaction in academia. The notion of “philoso-praxis” (Bangura, 2005), which refers to the practical application of philosophical principles, helps bridge Ubuntu's theoretical and action-based dimensions.

Ubuntu provides a transformative framework for promoting equity in academia. Its ethos, encapsulated in the phrase “*I am because we are*,” underscores the interconnectedness of individual identity and collective wellbeing (Mbigi and Maree, 1995; Venter, 2004). Eze (2008) critiques the notion that community is always prior to the individual, arguing instead that both co-constitute each other in a dynamic relationship. This challenges dominant interpretations of Ubuntu as demanding consensus at all costs, which can suppress diverse perspectives (Elonga Mboyo, 2019; Dillon and Pritchard, 2022; Muchineripi Kayange, 2020). Ramose (2004) emphasizes that Ubuntu is not merely an ethical framework but a challenge to the epistemic dominance of Western knowledge systems in education. He argues that the exclusion of African philosophy from curricula is a moral injustice that perpetuates colonial epistemologies. By embedding Ubuntu within academic structures, we move toward a more inclusive and representative system of knowledge production, one that values African intellectual traditions alongside Western paradigms.

In higher education, Ubuntu's collectivist philosophy stands in contrast to the competitive and individualistic norms that pervade academic environments (Letseka, 2012; Eyong, 2019). These norms often privilege solitary accomplishments, such as sole authorship and hierarchical structures, which disproportionately marginalize those without access to networks or mentorship opportunities. For Black Women, systemic barriers such as underrepresentation in leadership roles and exclusion from advancement opportunities exacerbate inequities. Ubuntu counters these barriers by fostering environments where collaboration and shared success are central (Collins, 2000; Jones, 2006).

Social capital, the networks and relationships that enable access to resources and opportunities, is a key factor in academic success (Bourdieu, 2011). However, systemic biases often impede Black Women from building these essential networks (Patton and Catching, 2009). Ubuntu provides a model for cultivating equitable networks that prioritize interconnectedness and mutual upliftment (Venter, 2004; Jones, 2006). Ubuntu-inspired frameworks empower Black Women to navigate academia through collective resilience and shared support. Practical applications include culturally relevant mentorship programs and peer networks grounded in Ubuntu, creating safe spaces where historically marginalized voices can thrive (Aronson and Laughter, 2016). Rather than enforcing conformity through consensus, Ubuntu prioritizes relational ethics, shared responsibility, and a commitment to equity, challenging dominant individualistic models.

### 1.2 The need for Ubuntu in the academy

Black Women in academia face multiple systemic barriers that extend beyond institutional gatekeeping (Collins, 2000; Patton and Catching, 2009). Ubuntu-inspired networks offer a transformative space where recognition is not contingent on institutional validation but on the collective affirmation of existence (Bangura, 2005). This aligns with the African cosmogram, which envisions relationships as circular engagements rather than linear transactions (Letseka, 2012).

Despite the growing emphasis on diversity, Black Women remain vastly underrepresented, accounting for only a small fraction of tenured faculty in predominantly White institutions (Collins, 2000; Patton and Catching, 2009). Compounding this disparity is exclusion from traditional mentorship and professional networks; this exclusion reinforces a cycle of inequity, limiting access to vital resources and support systems. Navigating predominantly White academic spaces presents challenges such as cultural isolation and microaggressions, contributing to the emotional labor required to assert one's presence. The absence of robust support systems exacerbates feelings of marginalization and emotional fatigue, negatively impacting both wellbeing and professional performance (Crenshaw, 1991; Fries-Britt and Turner Kelly, 2023).

Ubuntu-inspired networks provide pathways for solidarity, mentorship, and empowerment. These networks prioritize interconnectedness, dismantle hierarchical barriers, and create inclusive spaces where Black Women can thrive (Mbigi and Maree, 1995; Letseka, 2012).

## 2 Understanding Ubuntu-inspired networks

### 2.1 Definition

Ubuntu-inspired networks are relational structures grounded in the African philosophy of Ubuntu, which emphasizes the interconnectedness of all people, collective care, and the notion that “I am because we are” (Letseka, 2012; Mbiti, 1990). These networks are both a response to systemic marginalization and an alternative to hierarchical, individualistic models of academic engagement dominant in Western institutions. They function as culturally grounded grassroots spaces that center communal upliftment, reciprocity, and solidarity, particularly among marginalized groups such as Black Women in academia (Bangura, 2005; Ndlovu-Gatsheni, 2018a).

Unlike traditional professional networks, Ubuntu-inspired networks value relationality over competition and knowledge sharing over gatekeeping, offering support structures that are emotionally and intellectually sustaining (Chilisa, 2019). While they often emerge informally in response to exclusion from formal academic networks, they are no less strategic or impactful. These networks can take the form of mentorship collectives, affinity-based research groups, or race-conscious professional development communities. In all forms, they aim to disrupt exclusionary norms by centring culturally relevant values of care and connection. Recognizing these networks as legitimate and vital epistemic spaces is essential for transforming higher education into a more equitable and inclusive system (Le Grange, 2019; Makoe and Shandu-Phetla, 2019).

### 2.2 Characteristics

#### 2.2.1 Philosophically grounded in Ubuntu

Ubuntu-inspired networks represent a distinctive form of community-based knowledge production and support, underpinned by six interrelated characteristics that reflect their philosophical, structural, and functional commitments. First, these networks are philosophically grounded in the African humanist worldview of Ubuntu, which emphasizes the ontological interdependence of people, often encapsulated in the phrase “*I am because we are.”* Unlike generic grassroots collectives, Ubuntu-inspired networks are deeply rooted in principles such as relationality, collective humanity, and moral responsibility (Letseka, 2012). These principles are not merely abstract ideals but constitute a living ethic that shapes how members relate, collaborate, and organize. This grounding distinguishes Ubuntu networks from other grassroots formations by placing moral values at the heart of their design and intent, rather than treating them as incidental to their operation.

#### 2.2.2 Collective care and mutual support

Second, Ubuntu-inspired networks are characterized by a sustained commitment to collective care and mutual support. These networks prioritize emotional wellbeing, psychological safety, and reciprocal solidarity—particularly for Black Women and other marginalized individuals navigating exclusionary academic environments. The relationships forged within such spaces are built on compassion, trust, and dignity, reflecting a deliberate departure from the competitive, transactional ethos of many formal academic networks (Bangura, 2005; Makoe and Shandu-Phetla, 2019). Mutual care is not only a cultural imperative but also a strategy of survival and flourishing, offering members affirming spaces in which they can share vulnerabilities, exchange support, and collectively heal from institutional harm.

#### 2.2.3 Resistant to hierarchical and individualistic norms

Third, Ubuntu networks enact a resistance to hierarchical and individualistic norms, which are deeply entrenched in Western higher education institutions. By centring values such as collaboration, empathy, and community accountability, these networks function as counter spaces that oppose neoliberal and meritocratic paradigms (Le Grange, 2019). Rather than valorising individual achievement and productivity, Ubuntu-inspired collectives recognize that knowledge production and professional success are relationally achieved. In these spaces, success is redefined to include communal upliftment, shared growth, and ethical responsibility to others—values often obscured or devalued in dominant academic cultures.

#### 2.2.4 Community-centered knowledge exchange

Fourth, these networks facilitate community-centered knowledge exchange, informed by dialogical, non-hierarchical approaches to learning. Inspired by Ubuntu's emphasis on shared humanity, these spaces disrupt traditional notions of expertise and authority. Knowledge is shared horizontally, recognizing lived experience as equally valid as formal academic qualifications. This aligns closely with decolonial and Indigenous research methodologies, which reject the unidirectional flow of information from “experts” to “novices” and instead affirm co-created, contextually grounded epistemologies (Chilisa, 2019). Ubuntu-inspired networks thus act as critical epistemic spaces where alternative ways of knowing are validated and sustained.

#### 2.2.5 Informal yet intentionally structured

Fifth, while these networks are frequently informal in structure, they are intentionally organized and purposeful in function. They often exist outside formal institutional frameworks, operating with flexible, non-bureaucratic formats. However, their objectives—such as mentorship, racial and gender advocacy, and communal healing—are clearly defined and strategically pursued. The informality of these networks is thus a matter of structure rather than substance. They provide a strategic alternative for those excluded from institutional power, creating spaces of visibility and influence through culturally affirming and relationship-driven modalities.

#### 2.2.6 A subset of culturally specific grassroots networks

Finally, Ubuntu-inspired networks can be understood as a subset of culturally specific grassroots networks, which are diverse formations rooted in shared cultural, racial, or social identities. While they share many features with other grassroots formations, what sets Ubuntu networks apart is their explicit alignment with Ubuntu as a guiding ontology and praxis. Not all culturally specific grassroots networks draw upon Ubuntu; others may be grounded in Indigenous, feminist, or diasporic frameworks (Ndlovu-Gatsheni, 2018a; Chilisa, 2019; Collins, 2000). However, Ubuntu-inspired networks represent a distinct articulation within this broader category, offering a lens through which to reimagine higher education as a site of ethical interdependence and shared liberation.

## 3 Applying Ubuntu-inspired principles

### 3.1 Theory and practice

Ubuntu-inspired networks offer a transformative framework for fostering equity in academia through interconnectedness, mutual care, and collective progress. By challenging competition-driven academic models, these networks promote collaborative success and reciprocity (Mbigi and Maree, 1995; Cronshaw and Jackel, 2016).

These networks prioritize horizontal relationships, ensuring equitable access to resources and opportunities while fostering a sense of belonging (Letseka, 2012). For Black Women, who remain disproportionately underrepresented in academia, Ubuntu-inspired networks provide critical pathways for overcoming systemic inequities (Makoe and Shandu-Phetla, 2019; Asumah and Nagel, 2024).

### 3.2 Examples of Ubuntu-inspired practices

#### 3.2.1 Institutional support

Institutional support for Ubuntu-inspired practices involves more than surface-level diversity initiatives; it requires a deep, structural commitment to communal values, shared success, and collective upliftment. Grounded in the African-centered pedagogical model of Ubuntugogy, institutions are encouraged to rethink traditional hierarchies and adopt community-based, participatory models of learning (Bangura, 2005). Ubuntugogy embodies Ubuntu's principle that “*a person is a person through other people,”* positioning knowledge as relational, rather than transactional. When institutions embed these values into policy, such as revising recruitment, retention, and promotion criteria to reflect collective achievement, they move closer to realizing Ubuntu's transformative potential in academia (Letseka, 2012; Rasheem et al., 2018).

A compelling real-world example is the joint initiative by the Office for Students (OfS) and Research England (RE), which allocated nearly £8 million to 13 UK universities to address racial inequity in postgraduate research (UKRI, 2025). These projects aim to increase access and participation for Black, Asian, and minority ethnic students, fostering mentorship and equitable learning environments. This initiative reflects Ubuntu's communal ethos by redistributing resources, centring marginalized voices, and supporting systemic change. When institutional leadership champions such frameworks, it signals a meaningful commitment to structural inclusion rooted in shared humanity.

#### 3.2.2 Mentorship programs

Mentorship grounded in Ubuntu philosophy moves beyond hierarchical, transactional models to embrace relationships rooted in trust, reciprocity, and mutual growth. Ubuntu-based mentorship recognizes that personal and professional development occurs through connection, not isolation—“*I am because we are.”* For Black Women in academia, such mentorship offers a culturally affirming space for skill-building, emotional support, and navigating the structural barriers of predominantly white institutions (Fries-Britt and Turner Kelly, 2023; Allen and Joseph, 2018). These programs facilitate not only academic retention but also foster a sense of belonging and solidarity, reinforcing Ubuntu's principles of shared humanity and collective upliftment.

A strong example is the Shine Scholars Programme at the University of Surrey, which received £396,000 in funding to support Black British students across the academic pipeline (UKRI, 2025). The initiative offers mentorship, race equity training, internships, and fully funded PhD positions. By incorporating peer and reverse mentoring, teaching qualifications, and inclusive researcher development, the program fosters a rich environment for mutual learning and empowerment. It exemplifies Ubuntu in action—uplifting individuals through community, affirming cultural identity, and addressing systemic inequities by building networks of care, collaboration, and academic excellence.

#### 3.2.3 Peer networks

Peer networks grounded in Ubuntu philosophy play a vital role in supporting scholars, particularly Black, Asian, and minority ethnic women, through the often-isolating terrain of academia. These networks cultivate collective resilience by facilitating shared learning, emotional sustenance, and reciprocal collaboration. Rooted in the Ubuntu principle that one's wellbeing is intimately tied to the community, such networks provide safe spaces where members can collectively process experiences of marginalization, celebrate achievements, and navigate systemic challenges together (Fries-Britt and Turner Kelly, 2023; Assié-Lumumba, 2017; Bhambra et al., 2018; Thakhathi and Netshitangani, 2020).

An example is the Generation Delta project, led by six Black, Asian, and minority ethnic female professors and funded with £797,264. This initiative seeks to increase the number of racially minoritised women professors in English higher education by creating peer networks that support postgraduate researchers (PGRs) through key stages: access, retention, and career training. By addressing both individual and institutional barriers through mentorship, strategic advice, and capacity-building, Generation Delta enacts Ubuntu's ethos of shared upliftment and communal responsibility (UKRI, 2025). It affirms that transformation in higher education requires not only structural change but also peer-driven, culturally rooted communities of care and knowledge.

### 3.3 Application in context

While Ubuntu-inspired networks offer a robust framework for equity, relationality, and collective upliftment in higher education, their effectiveness is not universally guaranteed. The principles of Ubuntu—such as communal care, shared humanity, and mutual responsibility—must be adapted to the specific contexts in which networks operate. The Office for Students (OfS) and Research England funding initiative provides a valuable illustration of this. Although thirteen universities were awarded grants to improve access and participation for Black, Asian, and minority ethnic postgraduate researchers, the implementation and potential impact of each project vary considerably. The amount of funding allocated differs across institutions, with some receiving significantly more resources than others. This variability affects what each network can offer in terms of mentorship, support structures, and outreach capacity. Additionally, the strength of institutional partnerships, leadership buy-in, and infrastructural support all influence a project's long-term sustainability.

Geographic location is another critical factor. Projects based in more accessible urban centers may offer greater reach and participation, while those in geographically remote or less-connected cities may inadvertently limit access to the networks they aim to create. These disparities highlight that Ubuntu-inspired networks cannot be understood as fixed, standardized interventions. Instead, they must be seen as flexible, context-responsive models that embody Ubuntu principles in ways that align with local realities, resource availability, and community needs. Recognizing this ensures that the philosophy of Ubuntu is not reduced to a checklist but instead enacted thoughtfully and adaptively. Ultimately, the strength of an Ubuntu-inspired network lies not in uniformity, but in its ability to maintain core relational values while responding creatively and ethically to the diverse conditions within which it is situated.

While Ubuntu-inspired networks embody culturally rooted principles of care, relationality, and communal support, it is essential to recognize that no single network model is universally sufficient. Their grassroots and culturally specific nature provide vital safe spaces, primarily through race, gender, and cultural congruence. However, in striving for safety, such networks may unintentionally become exclusive, leaving some voices marginalized. Similarly, formal or broad-based networks may offer access to institutional resources and influence but can lack the intimacy and solidarity found in Ubuntu-informed spaces. The key lies not in privileging one model over another, but in recognizing that diverse networks serve different functions and intents. The super-objective—whether mentorship, advocacy, advancement, or healing—should guide the network's design and scope. Thus, building inclusive academic environments requires both culturally specific grassroots efforts and equitable formal structures, each contributing uniquely to the transformation of higher education.

## 4 Experiencing Ubuntu-inspired networks

In the following, we (the authors) reflect on our engagements with Ubuntu-inspired networks to illustrate how these frameworks have shaped our academic journeys. Through lived experiences, we explore the tangible impact of race-centered scholarly communities and culturally specific support systems rooted in Ubuntu values such as interconnectedness, mutual care, and shared humanity. These reflections highlight how Ubuntu principles are not only theoretical but lived and enacted through meaningful relationships, collective empowerment, and community resilience. Our narratives demonstrate the necessity of fostering inclusive academic spaces where culturally grounded, supportive networks are central to equity and systemic transformation.

### 4.1 Leveraging Ubuntu-inspired scholarly communities

A pivotal moment in my (Chiedza Ikpeh) academic journey was attending conferences organized by race-centered and anti-racism academic centers across the UK during my PhD years. These conferences, prioritizing Black and global majority representation, were transformative. Ubuntu's principles—interconnectedness, shared humanity, and mutual care—were not abstract concepts but lived experiences. For once, I was not a token minority; instead, I stood among peers, mentors, and scholars who shared similar contexts, experiences, and aspirations for racial equity in academia.

In academia's often-isolating environment, these conferences revealed the collective's power. They showed I was not alone in navigating systemic challenges like underrepresentation and exclusion. Ubuntu's ethos, “I am because we are,” became a tangible force, strengthening my resolve and anchoring me in a resilient community. These networks provided mentorship, collaboration, and inspiration, offering practical support and emotional reassurance.

The experience underscored the importance of spaces of belonging, grounded in Ubuntu principles, for the professional and personal growth of marginalized academics. Such spaces illuminate the value of solidarity in dismantling barriers and advancing equity. My recommendation is for institutions to prioritize and invest in these Ubuntu-inspired frameworks, ensuring they are integral to academic culture rather than exceptions. These networks do not just empower individuals; they enrich the entire higher education ecosystem, fostering inclusivity and collaboration essential for systemic transformation.

### 4.2 Cross-collaboration in Ubuntu-inspired networks

My (Iman Federico Awi) experience with support networks during my academic journey has demonstrated the transformative power of Ubuntu in addressing the unique challenges faced by Black Women in academia. I have participated in formal networks, which often prioritize institutional goals and broad diversity strategies. I have also had the opportunity to engage in culturally specific, Ubuntu-inspired networks, grounded in mutual care and shared experiences.

Formal networks can be beneficial for connecting diverse groups and are also very financially accessible. However, these networks, at times, have proven to be limiting, especially when their focus is on meeting funding requirements, understandably. The obligation to deliver measurable outcomes often leads to one-size-fits-all approaches that can fail to address the nuanced needs of Black Women. For example, broader networks may emphasize generic mentorship and support models that lack the cultural sensitivity to navigate the systemic barriers Black Women face in academia.

In contrast, by engaging with informal networks focused on the growth and development of Black Women, I have forged relationships built on trust, reciprocity and shared success. This sisterhood has provides us with a platform to give and receive emotional reassurance and practical advice tailored to our experiences as Black Women.

My recommendation for a critical step forward, is for institutions to facilitate partnerships between formal networks and Ubuntu-inspired networks. By creating collaborative platforms, institutions can leverage the targeted support provided by Ubuntu-inspired networks while addressing systemic issues through broader structural change. This collaboration can bridge gaps, ensuring all voices are heard and supported.

## 5 Conclusion

Ubuntu offers a framework for addressing systemic inequities in the academy, grounded in values such as communalism, solidarity, compassion, dignity, and relational interdependence (Letseka, 2012). These principles challenge the dominance of individualistic paradigms in Western higher education and instead advocate for a collective, human-centered approach. Ubuntu's transformative potential lies in its ability to create inclusive academic spaces where mutual care and shared humanity are central, and where marginalized voices—especially Black Women's—are not only heard but uplifted (Dillard and Neal, 2020).

A key takeaway is the critical role of networks in overcoming systemic inequities. These networks, rooted in Ubuntu values of collective responsibility and mutual support, offer Black Women avenues for mentorship, solidarity, and empowerment in academia. As demonstrated through theoretical reflections and lived experiences, Ubuntu-centered initiatives—such as peer-driven support groups and race-centered academic conferences—highlight the power of community to foster resilience, belonging, and professional growth. The above real-world examples illustrate how the abstract values of Ubuntu can be translated into concrete, actionable strategies.

The call to action is clear: institutions, policymakers, and academics must embrace Ubuntu as a guiding principle in their work. This involves not only creating policies that reflect Ubuntu's values but also committing resources to support networks and initiatives grounded in these ideals. Furthermore, ongoing research is essential to evaluate the long-term impact of Ubuntu-inspired frameworks on equity in higher education. By centring Ubuntu, academia can be reimagined as a space of mutual flourishing, where knowledge production and professional advancement are rooted not in competition, but in interconnectedness, compassion, and collective success.

### 5.1 Future directions in research and practice

#### 5.1.1 Implications for Black Women academics

Ubuntu-inspired networks hold transformative potential for Black Women in academia, offering culturally affirming spaces that foster resilience, collaboration, and career progression (Fries-Britt and Turner Kelly, 2023; Allen and Joseph, 2018). Future initiatives should focus on embedding Ubuntu values into formal academic development programs and leadership pathways to counteract systemic isolation and underrepresentation. Institutions must recognize the unique challenges Black Women face and prioritize sustained, culturally specific mentorship structures. Equitable access to research funding, peer networks, and institutional recognition of collective achievement are essential to ensuring that Black Women thrive within the academic hierarchy (Rasheem et al., 2018).

#### 5.1.2 Implications for Black Women professionals in HE

While often overlooked in equity discourse, Black Women in professional services roles encounter similar marginalization within institutional hierarchies. Future research and practice should explore how Ubuntu frameworks can be applied beyond faculty positions to create inclusive environments for Black professionals in administration, support, and governance. Ubuntu's emphasis on relationality and dignity supports the formation of collaborative, cross-functional networks that center wellbeing and shared purpose (Bangura, 2005). Institutions must adopt holistic inclusion models that uplift Black Women professionals, recognizing their labor and leadership as integral to systemic transformation (Dillard and Neal, 2020).

#### 5.1.3 Implications for Global South scholars in the academy

Ubuntu's African philosophical roots offer Global South scholars a powerful epistemic lens to reclaim space within predominantly Western academic systems. Future directions should include decolonising research metrics, amplifying Global South methodologies, and funding South-South collaborations (Ndlovu-Gatsheni, 2018b; Chilisa, 2019). Ubuntu-inspired networks can serve as platforms for knowledge exchange, advocacy, and solidarity, countering the dominance of Eurocentric paradigms. Institutions in the Global North must also invest in equitable partnerships that value reciprocal learning and epistemic justice. Embedding Ubuntu in global academic practices holds promise for a more just and inclusive scholarly ecosystem.
